# Prevalence and correlation of workload and musculoskeletal disorders in industrial workers: a cross-sectional study

**DOI:** 10.3389/fresc.2025.1677621

**Published:** 2025-09-23

**Authors:** Anderson G. Rodríguez-Pulido, Andy F. Arrieta-Córdova, Miguel A. Arce-Huamani

**Affiliations:** ^1^ Universidad Privada Norbert Wiener, Lima, Peru; ^2^ Universidad Privada Norbert Wiener, Vicerrectorado de Investigacion, Lima, Peru

**Keywords:** workload, musculoskeletal disorders, industrial workers, prevalence, occupational health, SDG 3: good health and well-being

## Abstract

**Background:**

Musculoskeletal disorders (MSDs) are a leading cause of disability among industrial workers worldwide, often resulting from excessive physical and mental workload. This study aimed to determine the prevalence of MSDs and their correlation with workload including physical and mental dimensions among industrial workers in the cleaning and ballasting division of a Peruvian shipyard.

**Methods:**

A cross-sectional, correlational study was conducted in 2023 among 100 workers selected from a population of 134. Workload was assessed using a validated questionnaire covering physical and mental dimensions, while MSDs were evaluated with the standardized Nordic Musculoskeletal Questionnaire. Descriptive statistics summarized the prevalence of workload and MSDs, and the relationship between variables was analyzed using Spearman's correlation coefficient, considering a significance threshold of *p* < 0.05.

**Results:**

High workload was identified in 85% of workers, with both physical and mental workload dimensions reaching high levels in the same proportion. The prevalence of severe MSDs was 88%, affecting primarily the wrist/hand, neck, shoulder, lumbar region, and elbow/forearm. A strong and statistically significant positive correlation was found between overall workload and MSDs (Spearman's rho = 0.896, *p* < 0.001). Similar correlations were observed for the physical (rho = 0.834) and mental (rho = 0.896) workload dimensions.

**Conclusion:**

Severe musculoskeletal disorders are highly prevalent among industrial workers exposed to substantial physical and mental workload. The strong correlations observed highlight the need for comprehensive occupational health strategies that address both ergonomic and psychosocial risk factors to reduce the burden of MSDs in industrial settings.

## 1 Introduction

Work-related musculoskeletal disorders (WMSDs) are a leading cause of occupational disability and functional loss worldwide, representing a major challenge for rehabilitation sciences, public health, and the sustainable well-being of workers (1). Globally, WMSDs account for millions of disability-adjusted life years and are among the most frequent occupational diseases, affecting diverse sectors and contributing to substantial economic and social costs (2). The prevalence of WMSDs among industrial workers is consistently high, with systematic reviews reporting rates exceeding 50% and highlighting the lower back, neck, and shoulders as the most affected anatomical regions (1). Industrial contexts are especially vulnerable due to intense physical and mental workload, repetitive tasks, and suboptimal ergonomic conditions that collectively undermine worker functionality and quality of life (3).

Recent studies also recognize that musculoskeletal health is shaped not only by biomechanical factors but by the cumulative burden of psychosocial stressors, workplace organization, and individual susceptibility, broadening the scope of prevention and intervention (4). The rehabilitation of work-related musculoskeletal injuries has thus become an urgent scientific and policy priority, directly supporting the aims of the Sustainable Development Goals (SDG 3: Good Health and Well-being) by fostering healthy lives and promoting well-being across all ages (5). Occupational rehabilitation, integrating biological, clinical, and socio-humanistic strategies, emerges as a crucial approach to optimize human functioning, sustain employability, and prevent chronic disability (6). Furthermore, ensuring safe and health-promoting work environments for industrial labor aligns rehabilitation efforts with a global agenda to reduce inequalities, strengthen workforce participation, and enhance the resilience of health systems (7). The implementation of participatory ergonomics, workload regulation, and workplace-based interventions has demonstrated effectiveness in reducing the risk and severity of WMSDs, particularly when adapted to local occupational settings (8). However, knowledge gaps persist regarding the interaction between physical and mental workload, unsafe behaviors, and the development of WMSDs, especially in diverse manufacturing environments (9).

Notably, evidence from Latin American and Asian industries indicates that effective occupational health management systems, rest periods, and ergonomic redesigns are still limited in practice, underscoring the need for context-specific, scalable solutions (4). In this regard, the promotion of occupational rehabilitation is fundamental not only for restoring function but also for preventing the long-term consequences of musculoskeletal injury among vulnerable worker groups (10). Despite these advances, there is a lack of integrative studies that simultaneously address the prevalence of WMSDs and their correlation with both physical and mental workload in industrial workers, using frameworks that reflect the multidimensionality of health, disability, and human functioning (7). In summary, WMSDs are a global health and rehabilitation challenge, closely linked to the goals of SDG 3 and demanding integrative occupational strategies that address their biological, clinical, and socio-humanistic dimensions.

Therefore, the objective of the present study was to determine the prevalence of musculoskeletal disorders and their correlation with workload (including physical and mental dimensions) among industrial workers.

## 2 Materials and methods

### 2.1 Study design and setting

This was a cross-sectional, correlational study conducted at the industrial shipyard SIMA-Chimbote, located in Ancash, Peru. Data collection took place between May and December 2023. The study was approved by the Institutional Ethics Committee of Universidad Privada Norbert Wiener (approval date: October 21, 2023). All procedures were performed in accordance with the ethical principles of the Belmont Report, ensuring voluntary participation, confidentiality, and anonymity throughout the study. Informed consent was obtained from all participants prior to data collection. The study adhered to the STROBE guidelines for observational studies in epidemiology (11).

To anchor the study context, we focused on a single, well-defined cohort industrial workers from the cleaning and ballasting division of the SIMA-Chimbote shipyard (Ancash, Peru) who routinely perform repetitive manual tasks and prolonged standing during standard 8-h shifts. This homogeneous context provides a clear exposure profile to relate both physical and mental workload to musculoskeletal outcomes; however, because objective records on rest breaks, shift scheduling, and enforcement of occupational standards were not collected, we do not attempt cross-industry comparisons and instead focus on within-cohort associations.

### 2.2 Participants

The target population comprised 134 workers from the cleaning and ballasting area of SIMA-Chimbote. Inclusion criteria were workers aged 20–50 years, full-time employment (8 h per day), and at least one year of service in the company. Exclusion criteria included administrative staff, vessel workers, workers from outside the company, and those not using personal protective equipment. Based on a simple random sampling strategy, a final sample of 100 eligible workers was selected and surveyed for this study. Individual demographic identifiers (including age and sex) were not collected by design to preserve worker anonymity within this small, single-division workforce; eligibility screening ensured the 20–50 years age range without recording individual ages. The sample size was determined using a 95% confidence level, 5% margin of error, and an assumed prevalence of 50% for musculoskeletal disorders, resulting in a calculated minimum sample of 100 participants.

### 2.3 Outcomes

The primary outcome was the prevalence and severity of musculoskeletal disorders (MSDs) among industrial workers. MSDs were assessed using the validated Standardized Nordic Musculoskeletal Questionnaire (NMQ) (12), which includes 11 items covering five anatomical regions: neck, shoulder, lower back, elbow/forearm, and wrist/hand. This instrument provides total and region-specific scores, classifying MSDs as mild (30–57 points), moderate (58–86 points), or severe (87–115 points). For each anatomical region, severity was also classified as mild (6–11 points), moderate (12–17 points), or severe (18–23 points). The NMQ was completed under supervised self-administration, and scoring followed established guidelines.

Workload was measured using a 31-item questionnaire adapted (13), validated and pilot-tested locally. This tool assesses both physical workload (16 items) and mental workload (15 items), with Likert-scale responses from 1 (“never”) to 5 (“always”) per item. The total workload score was classified as low (31–71 points), moderate (72–113 points), or high (114–155 points). Similarly, scores for physical and mental workload were categorized as low, moderate, or high according to established cutoffs. The questionnaire demonstrated high internal consistency (Cronbach's alpha > 0.95 in pilot testing).

Data were collected using supervised self-administered questionnaires on-site during working hours; trained research assistants were available for read-aloud and clarification upon request.

#### 2.3.1 Questionnaire administration and bias control

To minimize measurement bias during administration of the Nordic Musculoskeletal Questionnaire, we implemented supervised self-administration with standardized written instructions and a neutral script. Trained research assistants (blinded to study hypotheses) were available upon request to read items aloud, clarify wording using non-leading, preapproved phrases, and ensure understanding without suggesting answers. The validated Spanish version of the NMQ was used together with the standard body manikin to anchor anatomical regions. Before recording responses, assistants performed brief teach-back checks (participants paraphrased item meaning) when assistance was requested. We emphasized confidentiality and the absence of workplace consequences to reduce social desirability bias, and interviews were conducted in a quiet area away from supervisors. The NMQ recall windows (past 12 months; past 7 days) were reinforced with calendar cards to limit recall error. Field supervisors audited a random 10% of forms for completeness and internal consistency.

### 2.4 Statistical analysis

All data were analyzed using Stata version 18. Descriptive statistics were computed for all study variables, with categorical data summarized as frequencies and percentages and continuous variables reported as means and standard deviations where appropriate. The distribution of quantitative variables was assessed using both the Kolmogorov–Smirnov and Shapiro–Wilk tests (14), confirming significant deviations from normality (*p* < 0.001 for all main variables). Therefore, non-parametric statistical methods were applied. The association between overall workload and musculoskeletal disorders, as well as between the physical and mental workload dimensions and musculoskeletal disorders, was evaluated using Spearman's rank correlation coefficient (*ρ*) (15). The strength and direction of correlations were interpreted according to standard guidelines, and two-sided *p*-values below 0.05 were considered statistically significant (16). Analyses are unadjusted because demographic covariates (age and sex) were not collected by design. Cross-tabulations were used to further explore the relationship between workload levels and the severity of musculoskeletal disorders. All analyses were performed in accordance with the Strengthening the Reporting of Observational Studies in Epidemiology (STROBE) recommendations. All analyses underwent senior biostatistical oversight by a co-author to ensure reproducibility and appropriate model specification.

## 3 Results

A total of 100 industrial workers from the cleaning and ballasting area participated in the study. By design, demographic characteristics (including age and sex) were not collected; therefore, descriptive summaries by age or sex are not reported. Most participants reported high workload (85%), while moderate and low workload levels were observed in 7% and 8% of workers, respectively. The distribution was identical for both physical and mental workload dimensions, with 85% of workers experiencing high levels in each case. Regarding musculoskeletal disorders (MSDs), 88% of workers were classified as having severe symptoms, with only 4% and 8% exhibiting moderate or mild MSDs, respectively (Table 1).

**Table 1:** Levels of workload and musculoskeletal disorders among industrial workers.

Variable	Low (%)	Moderate (%)	High (%)
Overall workload	8.0	7.0	85.0
Physical workload	8.0	7.0	85.0
Mental workload	8.0	7.0	85.0
Musculoskeletal disorders severity	8.0	4.0	88.0

Values are expressed as percentages. The classifications of workload and musculoskeletal disorders (MSDs) were based on validated instruments. Most participants reported high workload (85%) and severe MSDs (88%).

Normality tests for all main study variables, including the total and regional MSD scores as well as workload indices, showed significant deviation from a normal distribution (Kolmogorov–Smirnov and Shapiro–Wilk *p* < 0.001 for all variables). Consequently, non-parametric methods were used for statistical analysis (Table 2).

**Table 2:** Normality tests for study variables (Kolmogorov–Smirnov and Shapiro–Wil).

Variable	Kolmogorov–Smirnov (D)	*p*-value (KS)	Shapiro–Wilk (W)	*p*-value (SW)	Normality
Neck dimension	0.530	<0.001	0.342	<0.001	No
Shoulder dimension	0.394	<0.001	0.669	<0.001	No
Lumbar/Back dimension	0.516	<0.001	0.411	<0.001	No
Elbow/Forearm dimension	0.393	<0.001	0.681	<0.001	No
Wrist/Hand dimension	0.399	<0.001	0.676	<0.001	No
Musculoskeletal disorders (TME total)	0.361	<0.001	0.634	<0.001	No
Physical workload	0.540	<0.001	0.225	<0.001	No
Mental workload	0.540	<0.001	0.225	<0.001	No
Total workload	0.540	<0.001	0.225	<0.001	No

All variables significantly deviated from the normal distribution based on both tests (*p* < 0.001). Therefore, non-parametric methods were applied for subsequent analyses.

Spearman's rank correlation analysis revealed a strong, positive association between overall workload and the severity of musculoskeletal disorders (*ρ* = 0.896, *p* < 0.001). Both physical workload (*ρ* = 0.834, *p* < 0.001) and mental workload (*ρ* = 0.896, *p* < 0.001) demonstrated significant positive correlations with MSDs. Notably, the highest correlation was observed with the mental workload dimension (Table 3 and Table 4).

**Table 3:** Correlation between overall workload and musculoskeletal disorders.

Evaluated relationship	Spearman's Rho (*ρ*)	*p*-value
Overall workload vs. musculoskeletal disorders	0.896	<0.001

Spearman's rho revealed a strong, positive, and statistically significant correlation between overall workload and MSDs (*ρ* = 0.896, *p* < 0.001).

**Table 4:** Correlation between workload dimensions and musculoskeletal disorders.

Workload dimension	Spearman's Rho (ρ)	*p*-value
Physical workload	0.834	<0.001
Mental workload	0.896	<0.001

Both physical and mental workload dimensions showed significant positive correlations with MSDs. The strongest association was found with mental workload (*ρ* = 0.896, *p* < 0.001).

Table 5 shows the cross-tabulation of workload level and the severity of musculoskeletal disorders among industrial workers. Among workers with high workload, 85% exhibited severe MSDs, while none reported mild or moderate symptoms. In contrast, mild (6%) and moderate (2%) MSDs were observed almost exclusively among those with low workload, and moderate workload was associated with a mix of mild (2%), moderate (2%), and severe (3%) symptoms. Overall, severe MSDs were highly concentrated in the high workload group, whereas lower workload levels were associated with milder symptoms.

**Table 5:** Cross-tabulation between workload level and MSD severity.

Workload level	Mild	Moderate	Severe	Total (%)
Low	6	2	0	8.0%
Moderate	2	2	3	7.0%
High	0	0	85	85.0%
Total	8	4	88	100.0%

Among workers with high workload, 85% experienced severe MSDs. In contrast, mild or moderate symptoms were more frequent in those with low or medium workload levels.

**Figure 1:**
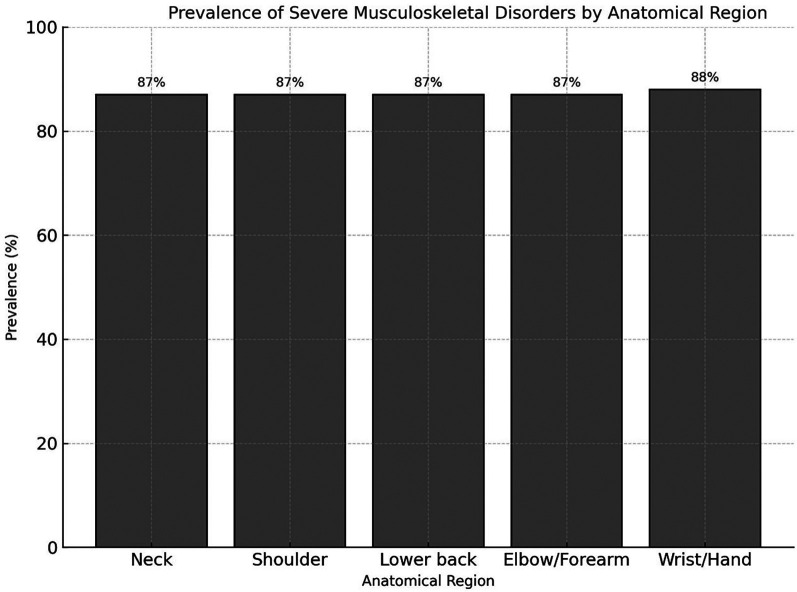
Distribution of severe musculoskeletal disorders by anatomical region among industrial workers. The wrist and hand region exhibited the highest prevalence (88%), followed closely by the neck, shoulder, lower back, and elbow/forearm (87% each).

Cross-tabulation showed that severe MSDs were highly prevalent among workers reporting high workload: 85% of participants with high workload also had severe MSDs, while mild or moderate symptoms were more frequent among those with low or moderate workload (Table 5). Analysis by anatomical region revealed that the wrist and hand exhibited the highest prevalence of severe symptoms (88%), followed closely by the neck, shoulder, lower back, and elbow/forearm, each with 87% (Figure 1).

## 4 Discussion

The findings of this study reveal an alarmingly high prevalence of musculoskeletal disorders among industrial workers exposed to substantial physical and mental workload. Nearly nine out of ten participants exhibited severe MSDs, with the majority also reporting high levels of both physical and mental occupational demands. The strong, positive correlations observed between workload particularly its mental dimension and MSD severity underscore the critical impact of psychosocial as well as ergonomic factors in this occupational group. These results indicate that, within the industrial context studied, heavy workload is not only widespread but is also closely intertwined with the severity of musculoskeletal complaints, highlighting a pressing need for integrated preventive and rehabilitative strategies in the workplace. The concentration of severe symptoms in workers facing high workload further supports the notion that the burden of occupational demands extends beyond physical strain, reinforcing the importance of a multidimensional approach to worker health and well-being.

The present study identified a remarkably high prevalence of severe musculoskeletal disorders (MSDs) among industrial workers, with 88% of participants affected. This figure stands out when contrasted with findings from international and regional literature. For instance, a comprehensive systematic review and meta-analysis of European industrial workers reported average MSD prevalences of 60% for the back, 54% for the neck/shoulder, and 42% for the wrist/hand, indicating a substantial burden but still lower than observed in our cohort (17). Similarly, in Brazilian industrial workers, clinical and imaging-based diagnoses of MSDs revealed a 100% prevalence of pain, with the shoulders and lumbar region most frequently affected (43.8% and 22%, respectively), again suggesting elevated but more region-specific rates (18). Additionally, studies of workers in intensive agricultural settings a comparable environment due to high physical demands reported an annual MSD prevalence of 50.9%, with lower rates of multi-region involvement (19). The notably higher prevalence in the present study may be explained by differences in occupational exposure, ergonomic risk, or definitions of severity applied, as well as potential underreporting in less rigorously surveilled settings. Importantly, these findings highlight the urgent need for comprehensive workplace interventions and policy action to address the overwhelming burden of severe MSDs in industrial sectors, underscoring the importance of strengthening occupational health strategies in similar contexts globally.

A strong association between overall workload and the occurrence of musculoskeletal disorders (MSDs) was observed in this study, as evidenced by the robust correlation found in our analysis. This finding aligns closely with international evidence, particularly the large-scale European meta-analysis, which identified general overload characterized by manual labor, repetitive movements, awkward postures, and excessive force as the principal risk factor for MSDs across various industrial sectors (17). Similarly, among Brazilian industrial workers, nearly all individuals diagnosed with MSDs were exposed to intensive manual tasks, with repetitive work present in 86.7% of cases and frequent overtime reported by 68.3%, clearly linking organizational and ergonomic overload with increased symptom severity (18). In another comparable setting, workers in greenhouse agriculture faced heightened MSD risk when physical inactivity and age over 40 were present, with odds ratios of 6.72 and 1.89, respectively, further supporting the role of cumulative workload and limited recovery in MSD pathogenesis (19). While methodological differences in how workload is defined and measured may partially account for variations in strength of association, the consistency of findings across diverse populations underscores the universal impact of total occupational load. These converging results reinforce the need for systematic interventions aimed at workload reduction and ergonomic optimization, with direct implications for occupational health policies across industrial environments.

The present study demonstrated a strong association between physical workload and the occurrence of musculoskeletal disorders (MSDs) among industrial workers, as reflected in a high correlation coefficient. This finding is in close agreement with previous research from various industrial settings. The European meta-analysis identified physical demands including manual tasks, load handling, awkward postures, and repetitive movements as the leading causes of MSDs, especially affecting the back and upper limbs (17). Similarly, in the Brazilian industrial cohort, most workers with MSDs were exposed to frequent handling of loads between 10 and 30 kg, repetitive actions, and prolonged standing, with trunk flexion and rotation consistently reported as relevant biomechanical risk factors (18). International risk assessments also highlight the impact of vibration exposure, use of hand tools, and uncomfortable working positions on the development of upper limb and lumbar disorders, such as osteoarthritis and lumbar discopathy, particularly in occupations like foundry and quarry work (20). Although specific workplace characteristics and ergonomic practices may vary, the converging evidence across industrial populations emphasizes that the physical demands of manual labor remain a critical and modifiable determinant of MSD risk. These insights strongly support targeted ergonomic interventions and workplace modifications as essential strategies for preventing physical workload-related MSDs, with relevance for occupational health standards worldwide.

In this study, a strong association was observed between mental workload and the prevalence of musculoskeletal disorders (MSDs) among industrial workers. This finding is in line with the growing recognition of psychosocial risk factors in the etiology of MSDs within occupational health literature. The European meta-analysis emphasized that work-related stress, pressure from accelerated work rhythms, monotony, and lack of recognition significantly increase the risk and severity of MSDs across industrial sectors (17). Comparable evidence from Brazilian industrial workers revealed that a majority perceived their tasks as monotonous (68.3%) and a substantial proportion described their work pace as “unbearable” (26.7%), with these organizational stressors strongly associated with higher pain reporting (18). Moreover, research on industrial nurses identified high mental demand and frustration scores measured via the NASA-TLX as significant predictors of increased MSD prevalence and severity, particularly among those with low job satisfaction (21). While the measurement of mental workload may differ across studies, the consistency of these findings highlights the central role of psychosocial stressors in the development and exacerbation of MSDs. Addressing mental workload through organizational change, stress management, and supportive work environments should therefore be prioritized within comprehensive occupational health interventions, with broad implications for the reduction of MSDs in industrial settings worldwide.

The anatomical distribution of severe musculoskeletal disorders (MSDs) observed in this study, with the highest prevalence in the wrist/hand, followed by the neck, shoulder, lumbar region, and elbow/forearm, aligns with trends reported in both international and regional literature. In the European meta-analysis, the most affected regions were the back (60%), neck/shoulder (54%), neck (51%), shoulder (50%), lumbar region (47%), wrist/hand (42%), and knee (33%), indicating a widespread pattern of multi-region involvement among industrial workers (17). Similarly, Brazilian industrial workers showed the greatest frequency of MSDs in the shoulders (43.8%) and lumbar spine (22%), with intervertebral disc alterations accounting for a significant proportion of clinical diagnoses (28.1%) (18). Studies in intensive agricultural settings also found that the lumbar region (36.2%), shoulder (19.5%), and knee (17.1%) were most affected, and a notable subset of workers (7.5%) reported symptoms in all examined regions (19). The consistent identification of upper limbs and the lumbar spine as key sites of MSDs across diverse industrial contexts suggests shared occupational exposures, such as repetitive tasks, forceful exertion, and prolonged static postures. Understanding these anatomical patterns is essential for tailoring ergonomic interventions and surveillance strategies, with the goal of reducing the burden of MSDs among industrial workers globally.

The results of this study demonstrate an exceptionally high prevalence of severe musculoskeletal disorders (MSDs) and strong associations with both physical and mental workload, which have significant implications for the design of workplace interventions and rehabilitation strategies. First, these findings underscore the urgent need for evidence-based rehabilitation programs that prioritize early detection, multidisciplinary management, and tailored exercise protocols to prevent chronic disability and optimize functional recovery among industrial workers. Ergonomic adaptation of workstations, task rotation, and regular breaks should be systematically implemented to address the high-risk anatomical regions identified, especially the upper limbs and lumbar spine, thereby reducing cumulative biomechanical stress. Consistent with international recommendations, such as those reported in European and Brazilian studies, integrating ergonomic education and fostering a culture of safety and self-care can enhance both short- and long-term worker well-being.

Beyond individual-level interventions, these results highlight the necessity of bridging the gap between research and clinical practice in occupational rehabilitation. By systematically documenting risk factors and outcome patterns, our study provides a robust evidence base to inform the development of context-specific, scalable programs, and supports the integration of rehabilitation expertise into occupational health policy. This aligns with international calls for multidisciplinary, preventive approaches and policy frameworks that address both physical and psychosocial dimensions of worker health, as emphasized in global literature. Implementing such comprehensive strategies can not only prevent disability and enhance functional outcomes but also contribute to sustainable productivity and improved quality of life for industrial workers worldwide.

Although this study offers valuable insights into the prevalence and correlations of musculoskeletal disorders (MSDs) among industrial workers, its generalizability should be considered with caution. The findings are based on a specific cohort from the cleaning and ballasting area of a single Peruvian shipyard, which may limit extrapolation to other industrial sectors or geographic contexts with different occupational exposures, ergonomic conditions, and health surveillance systems. Nonetheless, the high rates of severe MSDs and strong associations with both physical and mental workload observed here are consistent with global literature, suggesting that similar risk patterns may exist in comparable industrial settings across Latin America and beyond. To strengthen external validity, future studies should include diverse occupational groups and multinational samples, enabling broader policy translation and the development of internationally relevant occupational health interventions.

### 4.1 Strengths

This study leveraged validated instruments to capture both musculoskeletal outcomes (Nordic Musculoskeletal Questionnaire) and workload (physical and mental), and applied a pre-specified, appropriate non-parametric analysis given the non-normal distributions. A well-defined industrial cohort within a single shipyard division minimized exposure heterogeneity, and supervised self-administration with standardized instructions, neutral scripts, read-aloud on request, use of the body manikin, and brief teach-back checks improved comprehension and reduced interviewer and social-desirability bias. High response rates and strict inclusion criteria further enhance internal reliability.

### 4.2 Limitations

The cross-sectional design precludes causal inference, and focusing on one occupational subgroup in a single facility limits generalizability to other industries or regulatory contexts. Outcomes and exposures were self-reported, so information bias cannot be ruled out despite the safeguards; common-method variance and residual confounding by unmeasured ergonomic, organizational, or lifestyle factors may persist. Importantly, variability in health literacy could have affected item understanding in this industrial population; although we used supervised self-administration, read-aloud support, standardized clarifications, and teach-back, some misclassification is still possible. We did not collect objective data on rest breaks, shift patterns, or enforcement of occupational standards, which constrains cross-industry comparability and addresses the reviewer's concern about “acceptable conditions.” Future studies should include brief literacy screening, interviewer-administered modes when indicated, and objective ergonomic and scheduling measures to strengthen external validity and interpretability.

In summary, this study determined that the prevalence of severe musculoskeletal disorders among industrial workers was alarmingly high, with 88% of participants affected. Both physical and mental workload were strongly and positively correlated with MSD severity, with mental workload demonstrating the highest correlation. These findings highlight the urgent need for integrative occupational health and rehabilitation strategies that address not only the biomechanical but also the psychosocial demands of industrial labor. By documenting the extent and multidimensional correlations of MSDs in this population, the study directly informs the design of preventive interventions, workplace adaptations, and evidence-based rehabilitation programs, ultimately aiming to reduce disability and improve worker well-being in high-risk industrial environments.

In conclusion, this study determined that the prevalence of severe musculoskeletal disorders among industrial workers was alarmingly high, with 88% of participants affected. Both physical and mental workload were strongly and positively correlated with MSD severity, with mental workload demonstrating the highest correlation. These findings highlight the urgent need for integrative occupational health and rehabilitation strategies that address not only the biomechanical but also the psychosocial demands of industrial labor. By documenting the extent and multidimensional correlations of MSDs in this population, the study directly informs the design of preventive interventions, workplace adaptations, and evidence-based rehabilitation programs, ultimately aiming to reduce disability and improve worker well-being in high-risk industrial environments.

## Data Availability

The raw data supporting the conclusions of this article will be made available by the authors, without undue reservation.
